# Clinically severe *CACNA1A* alleles affect synaptic function and neurodegeneration differentially

**DOI:** 10.1371/journal.pgen.1006905

**Published:** 2017-07-24

**Authors:** Xi Luo, Jill A. Rosenfeld, Shinya Yamamoto, Tamar Harel, Zhongyuan Zuo, Melissa Hall, Klaas J. Wierenga, Matthew T. Pastore, Dennis Bartholomew, Mauricio R. Delgado, Joshua Rotenberg, Richard Alan Lewis, Lisa Emrick, Carlos A. Bacino, Mohammad K. Eldomery, Zeynep Coban Akdemir, Fan Xia, Yaping Yang, Seema R. Lalani, Timothy Lotze, James R. Lupski, Brendan Lee, Hugo J. Bellen, Michael F. Wangler, Members of the UDN

**Affiliations:** 1 Department of Molecular and Human Genetics, Baylor College of Medicine, Houston, TX, United States of America; 2 Jan and Dan Duncan Neurological Research Institute, Texas Children's Hospital, Houston, TX, United States of America; 3 Baylor-Hopkins Center for Mendelian Genomics, Department of Molecular and Human Genetics, Baylor College of Medicine, Houston, TX, United States of America; 4 University of Oklahoma Health Sciences Center, Oklahoma City, OK, United States of America; 5 Nationwide Children’s Hospital & The Ohio State University, Columbus, OH, United States of America; 6 Department of Neurology and Neurotherapeutics, UT Southwestern Medical Center andTexas Scottish Rite Hospital, Dallas, TX, United States of America; 7 Houston Specialty Clinic, Houston, TX, United States of America; 8 Department of Pediatrics, Baylor College of Medicine, Houston, TX, United States of America; 9 Texas Children’s Hospital, Houston, TX, United States of America; 10 Howard Hughes Medical Institute, Houston TX, United States of America; Stanford University School of Medicine, UNITED STATES

## Abstract

Dominant mutations in *CACNA1A*, encoding the α-1A subunit of the neuronal P/Q type voltage-dependent Ca^2+^ channel, can cause diverse neurological phenotypes. Rare cases of markedly severe early onset developmental delay and congenital ataxia can be due to *de novo CACNA1A* missense alleles, with variants affecting the S4 transmembrane segments of the channel, some of which are reported to be loss-of-function. Exome sequencing in five individuals with severe early onset ataxia identified one novel variant (p.R1673P), in a girl with global developmental delay and progressive cerebellar atrophy, and a recurrent, *de novo* p.R1664Q variant, in four individuals with global developmental delay, hypotonia, and ophthalmologic abnormalities. Given the severity of these phenotypes we explored their functional impact in *Drosophila*. We previously generated null and partial loss-of-function alleles of *cac*, the homolog of *CACNA1A* in *Drosophila*. Here, we created transgenic wild type and mutant genomic rescue constructs with the two noted conserved point mutations. The p.R1673P mutant failed to rescue *cac* lethality, displayed a gain-of-function phenotype in electroretinograms (ERG) recorded from mutant clones, and evolved a neurodegenerative phenotype in aging flies, based on ERGs and transmission electron microscopy. In contrast, the p.R1664Q variant exhibited loss of function and failed to develop a neurodegenerative phenotype. Hence, the novel R1673P allele produces neurodegenerative phenotypes in flies and human, likely due to a toxic gain of function.

## Author summary

Calcium channels control the levels of calcium within cells and are important in human health. Indeed, groups of patients with disorders of balance known as ataxia have been found to have mutations in a calcium channel gene in the human genome called *CACNA1A*. *CACNA1A* mutations have also been observed in patients with particular forms of migraine leading to temporary paralysis on one side of the body (hemiplegia). Mutations in *CACNA1A* are increasingly found in even more severe brain phenotypes in childhood. This research focused on a group of 5 patients with that particularly severe *CACNA1A*-related disease. One of the patients had a particular genetic misspelling in *CACNA1A* while the other four had nearby misspellings. We used the fruitfly, *Drosophila melanogaster*, to generate flies with these same misspellings in a genetic background that lacked the fly version of the calcium channel. Interestingly, by studying these flies we saw differences between the mutation in Patient 1 and the other four patients. These differences suggest one of the mutations produces more neurodegeneration, and indeed we see more degeneration in that patient. The fly studies allowed us to understand the function of the mutations in these patients, and were helpful in guiding treatment decisions.

## Introduction

Voltage-gated calcium channels (VGCCs) are a family of calcium ion selective proteins that both mediate calcium entry into neurons at synapses upon depolarization and are required for calcium-dependent functions in the cell [1, 2]. VGCCs are composed of multiple subunits, including the α1 subunit which is encoded by the *CACNA1A* gene and is responsible for Ca^2+^ entry in neurons. Across species the α1 subunit is required in the nervous system. In mice the tottering (*tg*) mutants have mutations in *Cacna1a* and exhibit ataxia, motor seizures and cerebellar degeneration [3]. In *Drosophila* the channel is required for synaptic transmission and *cacophony (cac)* null alleles are lethal [4, 5]. In mosaic clones mutations in *cacophony* produce neurodegeneration with defective lysosomal fusion with autophagosomes [6].

Given the essential role of VGCCs in the nervous system it is not surprising that mutations in *CACNA1A* cause a spectrum of neurological disorders in humans[7]. Spinocerebellar ataxia type 6 (SCA6, OMIM #183086) is characterized by ataxia, dysarthria, and dysphagia with onset typically in middle adulthood but ranging from 20 to 60 years of age[8]. SCA6 is due to heterozygous expansions of CAG repeats within *CACNA1A* resulting in large polyglutamine tracts within the protein which cause cytoplasmic aggregations and Purkinje cell degeneration [9, 10]. Episodic ataxia type 2 (EA2, OMIM #108500) is characterized by episodes of ataxia with onset in childhood or early adulthood, and affected individuals are often responsive to carbonic anhydrase inhibitors such as acetazolamide [11–13]. EA2 is due to heterozygous deletions, stop-gains, frameshifts, or missense mutations [11, 14, 15]. Functional analysis based on patch-clamping suggests that they correspond to loss of channel function and hence reveal haploinsufficiency of the locus [16, 17]. While EA2 is typically episodic, many patients also have progressive ataxia due to ongoing neurodegeneration which is generally slow [15]. Familial hemiplegic migraine (FHM, OMIM # 141500) is a form of migraine with aura and transient hemiplegia and an age of onset between 5 years and early adulthood [18]. *CACNA1A* variants associated with FHM are typically missense alleles [19], and in contrast to EA2-associated missense alleles, electrophysiological evidence suggests that they are gain-of-function mutations [20]. FHM mutations appear to lead to hyperactivity of the channel [21] both by altering the biophysical properties as well as decreasing the inhibitory G-protein association with the channel [22].

While the disease phenotypes for EA2 and FHM appear to relate to different underlying genetic mechanisms, considerable phenotypic overlap between FHM and EA2 means that some individuals exhibit features of both disorders [23, 24]. Indeed even within the same family, the same *CACNA1A* variants can produce phenotypes more similar to FHM than EA2 [23, 25]. Several *de novo* missense alleles in *CACNA1A* have been reported in children with congenital ataxia and intellectual disability [26], as well as with non-progressive congenital ataxia with seizures[27]. However, channel function and its relation to phenotype are not well studied in these severe ataxias [26–28]. Most previous studies used *in vitro* electrophysiological analyses to assess channel function [20, 21, 29], leaving a need for functional annotation of *CACNA1A* and its variants in a model organism.

## Results

### Human subjects

We ascertained 5 individuals who underwent exome sequencing for global developmental delay and congenital ataxia, in whom *de novo* missense variants in *CACNA1A* were discovered. Patient 1 was enrolled in the Undiagnosed Diseases Network (UDN) at Baylor College of Medicine and concurrently in the Baylor-Hopkins Center for Mendelian Genomics (BHCMG) [30] as part of a large-scale research re-analysis of clinical exomes [31]. Patients 2–5 were identified at Baylor Genetics Laboratories (BGL) [32, 33]. All families gave written consent for exome sequencing.

The clinical findings of these individuals are summarized in **Table 1 (S1 Case Histories)**. All individuals (5/5) exhibited global developmental delay, expressive language delay and dysarthric (4/5) or no expressive speech (1/5; Patient 1). Interestingly all subjects had ataxia (5/5) but to varying degrees, with independent ambulation and unsteady gait in some (3/5) and more severely impaired ambulation requiring use of walker in others (2/5). Other neurological features such as behavioral problems, sensory processing disorders, aggressive behavior and attention deficit were also noted, although these features were not consistent among the patients.

**Table 1:** Clinical features of the 5 individuals with *de novo CACNA1A* variants.

Identifier	Patient 1	Patient 2	Patient 3	Patient 4	Patient 5
Coding variant (NM_001127221.1)	c.5018G>C	c.4991G>A			
Protein variant	p.R1673P	p.R1664Q			
Inheritance	*De novo*	*De novo*	*De novo*	*De novo*	*De novo*
Parental ages	38(mat), 38(pat)	17(mat), 20(pat)	30(mat), 33(pat)	18(mat), 22(pat)	38(mat), 41(pat)
Sex	Female	Female	Male	Male	Female
Age	8 years	5 years	5 years	8 years	6 years
Ancestry	Hispanic & European	Hispanic	European	Hispanic	European, Ashkenazi Jewish
Ataxia	Present; uses walker	Severe—independent ambulation difficult	Wide-based gait, frequent falls, significant toe walking, wears AFOs, uses adaptive stroller	Unsteady gait	Poor coordination, truncal ataxia
Cerebellar findings	Progressive cerebellar atrophy	None	None	Mild atrophy of cerebellar vermis, hemispheres normal	None
Other MRI findings	Mild thinning of the body and splenium of the corpus callosum; mild delay in deep white matter myelination	Normal MRI	Thin corpus callosum posteriorly	None	Normal MRI at 24m and 46m
Development	Global delays; no language (uses signs & iPad)	Global delays, has very few words	Global delays; walked at 27m, speech at 15m; dysarthric speech	Global delays; uses 6-7-word sentences; dysarthric; below normal IQ	Global delays (Expressive language delay, gross/fine motor delay); dysarthria, dysgrammatic speech
Hypotonia	Congenital, generalized	Some	Present	Present	Mild
Additional neurologic findings		Worsening behavioral issues; hyperreflexia	Difficulty sleeping, sensory processing disorder	Aggression	ADD/executive dysfunction; hyporeflexic
Ophthalmologic findings	Accommodative esotropia, hyperopia	Eye movement disorder	Strabismus, myopia, astigmatism	Ocular apraxia	Alternating strabismus, esotropia
Weight (percentile)	19th	90th	90th	89th	
Height (percentile)	25th	70th	82nd	51st	
Head circumference (percentile)	76th	75th	65th	78th	
Dysmorphisms	A tented upper lip and prominent jaw	None	Mild midface hypoplasia, occipital flattening, corrected plagiocephaly		None
Additional features		Constipation	Gastroesophageal reflux disease	Hyperextensible, inverted nipples	Low CSF HIAA, hyperextensible

ADD, attention deficit disorder; CSF, cerebrospinal fluid; HIAA, Hydroxyindoleacetic acid; m, months; mat, maternal age; pat, paternal age

**Fig 1:**
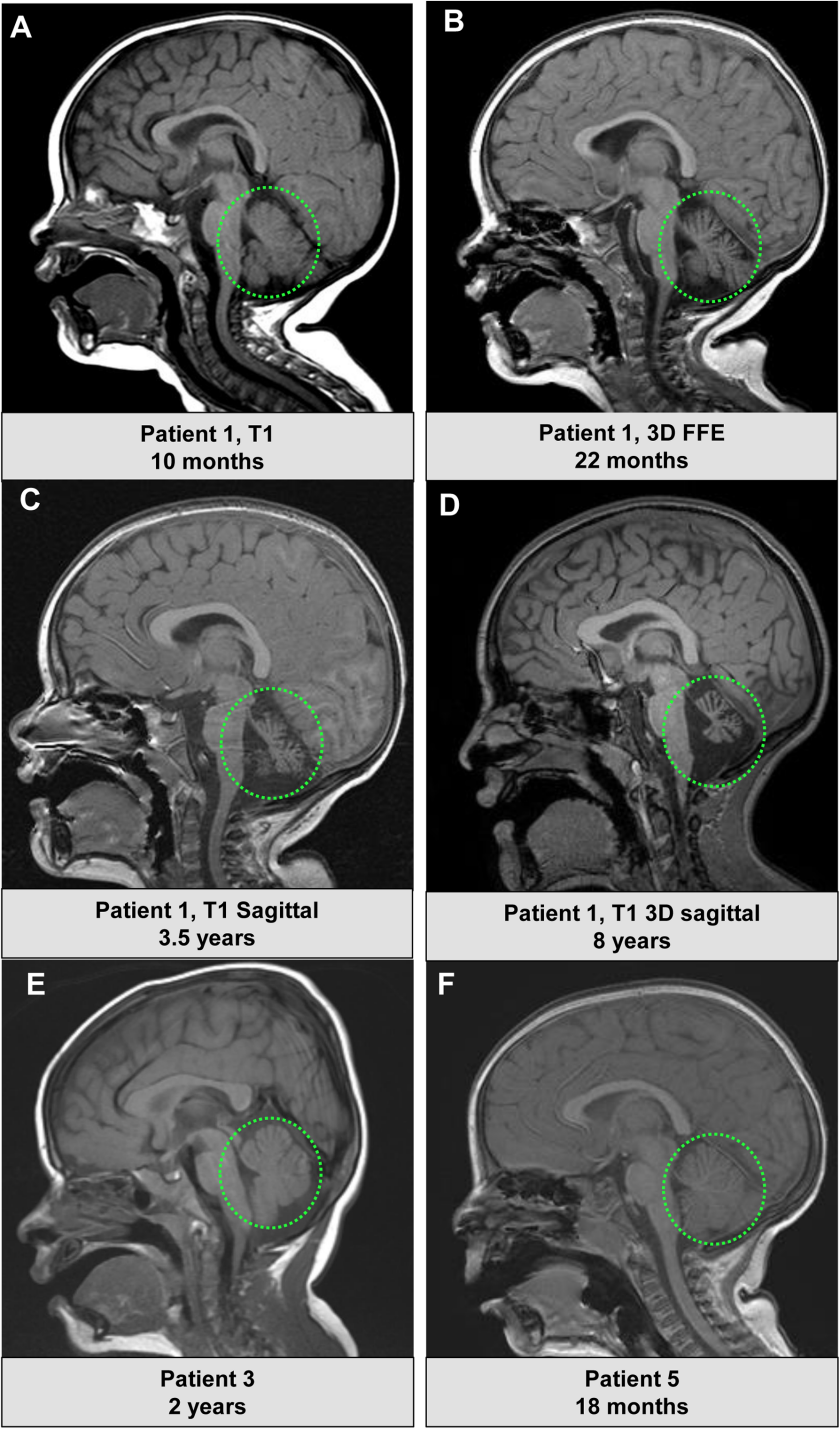
Neuroimaging characteristics of the patients with *CACNA1A de novo* variants. A) A T1 sequence showing an approximately mid-sagittal image of Patient 1 at 10 months of age. The cerebellum appears normal in size. B) Patient 1, a 3D FFE image at 22 months again showing a mid-sagittal view, with some increased prominence of the cerebellar folia suggesting early cerebellar atrophy C)Patient 1, T1 mid-sagittal view at 3.5 years showing progressive cerebellar atrophy D)Patient 1, a mid-sagittal T1 3D image at 8 years showing the neurodegenerative progression affecting the cerebellum E) Patient 3, a mid-sagittal T1 image at age 2 years, with normal cerebellum and thin corpus callosum posteriorly F) Patient 5, a mid-sagittal T1 image at age 18 months, normal.

Neuroimaging for the patients differed; one of the five subjects had evidence of progressive cerebellar atrophy. In Patient 1, the initial MRI at 10 months showed a normal cerebellum (**Fig 1A**), while imaging at 22 months revealed mild cerebellar atrophy (**Fig 1B**), which progressed at 3.5 years (**Fig 1C**) and 8 years (**Fig 1D**). Patient 1 is the only subject with progressive cerebellar degeneration. In Patient 3, the cerebellum appeared normal in size at 2 years, although the corpus callosum was thin posteriorly (**Fig 1E**). In Patient 5 the MRI is normal (**Fig 1F**). In Patient 4 there is some cerebellar hypoplasia involving the vermis (**S1A Fig**) but not the lobes of the cerebellum (**S1B Fig**)

### Whole exome sequencing

Trio-based exome sequencing for Patient 1 through BHCMG [31] revealed a *de novo* missense variant (NM_001127221:c.5018G>C: p.R1673P; chr19:13346480C>G [hg19]) in *CACNA1A* (**S1C Fig**). Patients 2–5 had clinical proband exome sequencing at BGL [32, 33] (see Materials and Methods) and were all found to have a recurrent missense *de novo CACNA1A* variant (NM_001127221:c.4991G>A: p.R1664Q; chr19:13346507C>T [hg19]) (**S1C Fig**). Notably, the four individuals with p.R1664Q and the single individual with p.R1673P all exhibit some similarities to a child previously reported with a *de novo* p.R1664Q allele with early onset ataxia without seizures or migraine[34].

**Fig 2:**
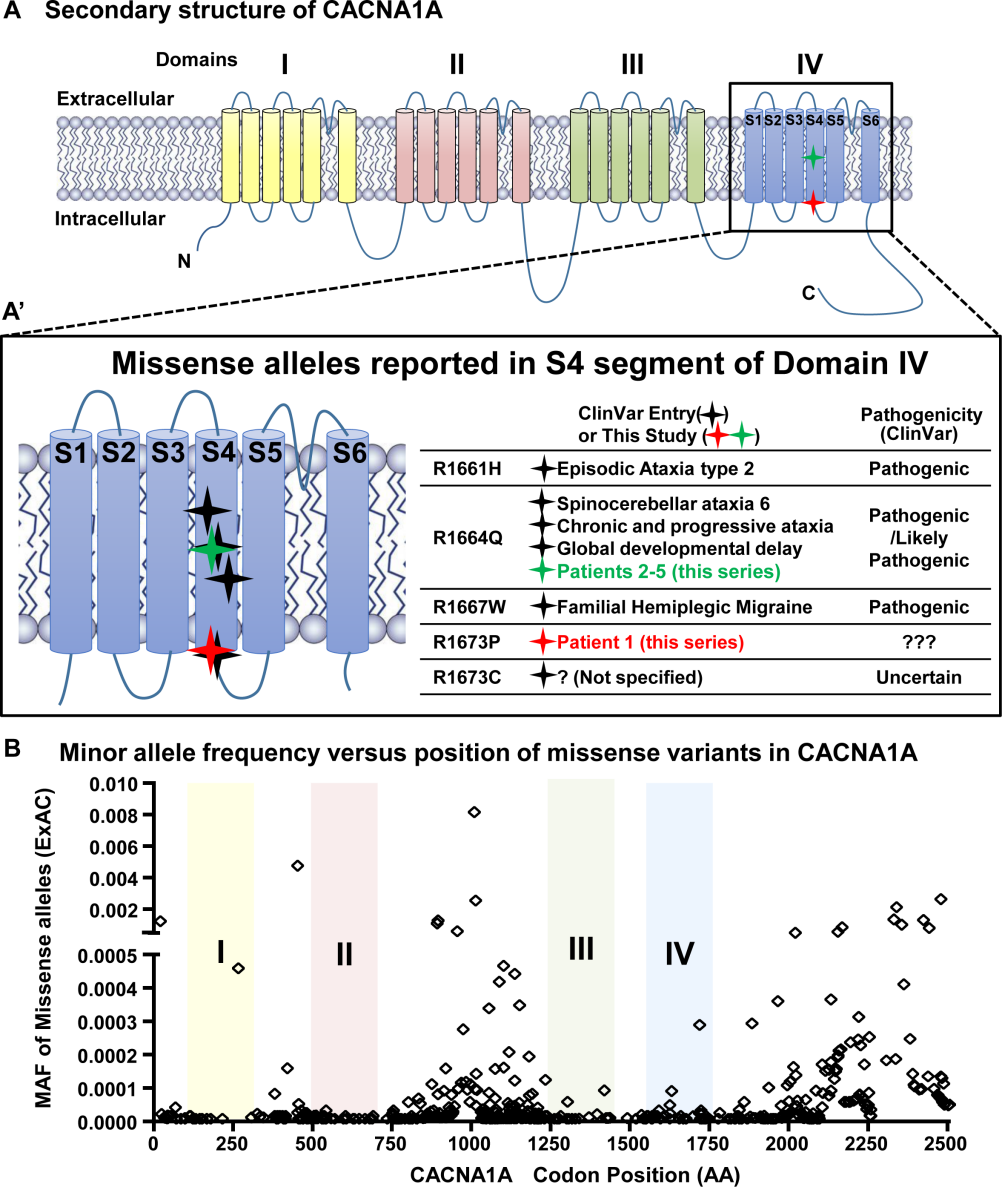
*CACNA1A de novo* variants affecting the S4 transmembrane segment of domain IV. A graphical representation of the overall structure of the α1 subunit of the voltage gated calcium channel encoded by *CACNA1A*. **A)** Both the R1673P (red star) (Patient 1) and R1664Q (green star) (Patients 2–5) variants occur in the S4 transmembrane segment of domain IV. Missense alleles from ClinVar in the S4 transmembrane segment of domain IV are shown as black stars. Missense changes in this segment appear associated with Episodic Ataxia 2 (p.R1661H), familial hemiplegic migraine (p.R1667W) and the severe congenital ataxia in patients in our series (p.R1664Q and p.R1673P). The p.R1664Q (Patients 2–5) is listed in ClinVar as associated with SCA type 6, chronic and progressive ataxia and global developmental delay. While the p.R1673P variant is novel, ClinVar does list p.R1673C as a variant of uncertain significance. **B)** Minor allele frequency for missense variants listed in the ExAC browser plotted according to the codon position. Only alleles with MAF <0.01 are plotted. Missense alleles appear rarely or with very low allele frequency within the four transmembrane domains of the CACNA1A protein. *CACNA1A* is intolerant to loss-of-function variation with 4 such variants observed in ExAC over 78.3 expected (probability of loss-of-function intolerance = 1.0)[53]. Furthermore, the pattern of missense variation in ExAC across the coding region of *CACNA1A* is consistent with paucity of variation within the transmembrane segments.

For each case the ratio of variant reads to total reads and the Sanger confirmation suggested approximately 50% variant alleles meaning the patients are heterozygous. Both *de novo* changes occur within CpG dinucleotides, hypermutable sites prone to methylation and deamination leading to *de novo* events [35–37]. These have been noted to affect arginine residues in a number of disease contexts [38–40]. The *de novo* missense changes in these patients affect conserved arginine residues at the S4 transmembrane segment of domain IV of the protein (**Fig 2A and 2A’**). Several pathogenic alleles associated with a range of phenotypes are reported to affect this transmembrane segment of domain IV in ClinVar [41] (**Fig 2A’).** For example, p.R1661H is associated with EA2, while p.R1667W is associated with FHM. The R1664Q allele seen in our patients 2–5 is associated with ataxia and global developmental delay. We observed that the intervals of the transmembrane domains (I-IV) all appear intolerant to missense variation (**Fig 2B**).

### *Drosophila cac* mutants and *Drosophila* transgenics

**Fig 3:**
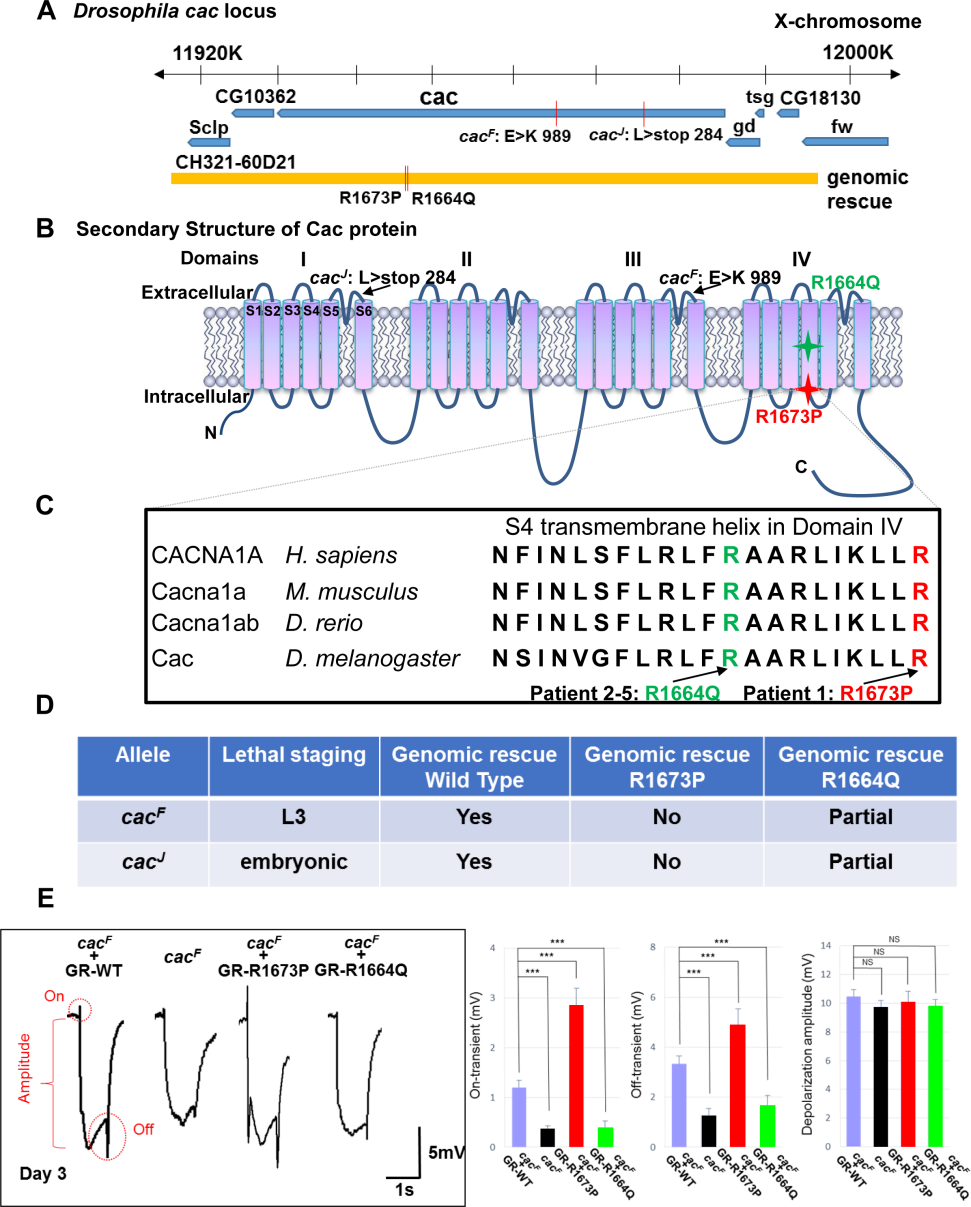
*Drosophila* lethality rescue experiments testing the *de novo CACNA1A* variants affecting the S4 transmembrane segment of domain IV. A) The *Drosophila cac* genomic context showing the P[acman] clone CH321-60D21. B) The membrane topology of the voltage gated channel showing the *cac* alleles used in this study and the position of the human variants that were affecting conserved residues engineered into transgenes. C) Protein alignment of CACNA1A and its homologs in mouse, *Drosophila* and zebrafish, showing the strong conservation of the entire S4 segment of domain IV. D) Lethality rescue experiments in *Drosophila* for *cac* lethal alleles by wild type, R1673P or R1664Q mutant genomic transgenes. Lethality rescue experiments in *Drosophila* were done by crossing *y w*, *cac*^*F*^ *FRT19A/FM7c or y w*, *cac*^*J*^ *FRT19A/FM7c* with *y w/Y; Dp(1;3)DC131(or -R1673P or -R1664Q) flies*. The males that have no *FM7c* marker in the next generation were considered rescued flies. The numbers of rescued flies were also compared with *FM7c* males in the same progeny. The rescue results are as follows: GR-WT for *cac*^*F*^: Rescued males (115) / *FM7c* males (67) = 1.72. GR-WT for *cac*^*J*^: Rescued males (90) / *FM7c* males (56) = 1.61. GR- *R1673P* for *cac*^*F*^: Rescued males (0) / *FM7c* males (86) = 0. GR- *R1673P* for *cac*^*J*^: Rescued males (0) / *FM7c* males (81) = 0. GR- *R1664Q* for *cac*^*F*^: Rescued males (50) / *FM7c* males (71) = 0.71. GR- *R1664Q* for *cac*^*J*^: Rescued males (41) / *FM7c* males (67) = 0.61. E) Electroretinograms of 3-day-old flies. An ERG trace consists of an amplitude (red bracket), an on-transient and an off-transient (red dotted circles). Quantifications of the on-transients and off-transients of ERG traces are on the right. Data are presented as mean ± SEM. p values were calculated using Student’s t test. ***p < 0.001; NS, not significant. p-values were consistent with those on one way ANOVA. In 3-day-old *cac*^*F*^ mutant clones carrying a wild type 80 kb P[acman] genomic rescue transgene (GR-WT) the electroretinogram has a normal pattern of synaptic activity (demonstrated by on- and off-transients) and depolarization (amplitude marker). In photoreceptors from *cac*^*F*^ mutants there is a loss of “on” and “off.” In the *cac*^*F*^ mutants with the mutant transgenes (GR-R1673P, GR-R1664Q) there are distinct findings with R1673P producing larger “on” and “off” transients while the R1664Q produces a loss-of-function phenotype similar to the *cac*^*F*^ mutants.

Notably these S4 arginine variants in domain IV display diverse phenotypes, and other severe cases reported also carry missense variants within the S4 segments in domains III [26, 27, 42] and IV [34] of *CACNA1A*. However, these mutations have not been modeled *in vivo*, and the functions of these missense variants are therefore not defined (i.e., haploinsufficient loss or gain of function). As drugs are available to either boost or inhibit calcium channel function, knowledge of mutation mechanism may have important implications for patients. In *Drosophila cacophony (cac)*, the homolog of *CACNA1A*, is required for synaptic transmission and lysosomal fusion, and *cac* null alleles are embryonic lethal [4, 5]. Previously, we isolated numerous alleles of *cac* in a forward genetic screen for essential genes that affect the function of photoreceptors based on defective ERGs in homozygous mutant eye clones [20]: *cac*^*J*^ is an early nonsense mutation which is an embryonic lethal, and *cac*^*F*^ is a missense mutation affecting a key glutamate residue in the calcium ion selectivity filter loop and is larval lethal (**Fig 3A and 3B**). In mosaic eye clones, both mutations lead to expanded nerve terminals, synaptic vesicle accumulations, and aberrant lysosome-autophagosome fusion defects [6].

To test the functional consequences of the variants identified in the five subjects, we designed a rescue-based strategy. To rescue the phenotypes associated with the *cac* alleles, we first selected a 77 kb P[acman] transgenic construct [6] that contains the entire 53 kb genomic region of *cac*, including endogenous enhancers to drive proper expression of the transgene. We introduced the two variants found in the subjects by recombineering and we inserted these genomic rescue (GR) constructs into the identical VK37 docking site [6] in the fly genome by phiC31-mediated recombination [6] to avoid position effects (**Fig 3A**). We labeled these constructs GR-WT (fly wild type), GR-R1673P (Patient 1) and GR-R1664Q (Patients 2–5) corresponding to the human proteins/variants. These mutations are within the S4 transmembrane segment of domain IV that is nearly identical between flies and humans (**Fig 3C**).

The wild type P[acman] transgene rescues the lethality associated with *Drosophila cac*^*J*^ and *cac*^*F*^ (**Fig 3D).** However, GR-R1673P (Patient 1) mutation failed to rescue lethality, whereas GR-R1664Q (Patients 2–5) was able to rescue lethality partially (41% of expected viable progeny). This data suggest that the two mutations have functional consequences *in vivo*, and that the R1673P mutation seems to behave as a more severely impaired allele.

### Electroretinograms reveals differential effects in *CACNA1A* alleles

Next we performed ERGs [6] in *cac* mutant clones rescued with either a wild type or a mutant P[acman] GR construct. ERG recordings reveal two key features: the ‘on’ and ‘off’ transients (red dotted circles, **Fig 3E**) typically reflect synaptic transmission between pre- and post-synaptic cells, whereas the amplitude of the depolarization (red bracket in **Fig 3E**) is a measure of the phototransduction activity. *cac* mutations typically affect the ‘on’ and ‘off’ transients but have little or no effect on the amplitude, consistent with the role of *cac* in synaptic vesicle release [6]. To assess the function of the variants from the subjects, we generated homozygous mutant eye clones of *cac*^*J*^ and *cac*^*F*^ in young animals (3 days old) and compared the ERGs in flies that carry the wild type and mutant P[acman] GR constructs. Both *cac* alleles exhibit loss of synaptic transmission as evidenced by loss of the ‘on’ and ‘off’ transients (**Fig 3E, S2A Fig**). Mutant flies carrying the wild type P[acman] *cac* transgene exhibited normal ‘on’ and ‘off’ transients (**Fig 3E, S2A Fig**). Interestingly, GR-R1673P (Patient 1) dramatically increased the amplitude of the on and off transients, whereas GR-R1664Q (Patients 2–5) failed to rescue the synaptic transmission defect caused by the *cac*^*J*^ and *cac*^*F*^ mutations. These data suggest that the R1673P allele seen in Patient 1 is a gain-of-function mutation, and R1664Q found in Patients 2–5 is a loss-of-function allele based on the ERG defects observed in the photoreceptors in *Drosophila*. We also examined the ‘on’ and ‘off’ transients of flies with the P[acman] transgenes in a wild-type *y w* (*cac +/cac+*) background as well as heterozygous backgrounds (*cac*^*J*^ /+ and *cac*^*F*^ /+). Although there was a slight nominal increase in the ‘on’ transient in the R1673P animals, it was not statistically significant (S2B Fig).

**Fig 4:**
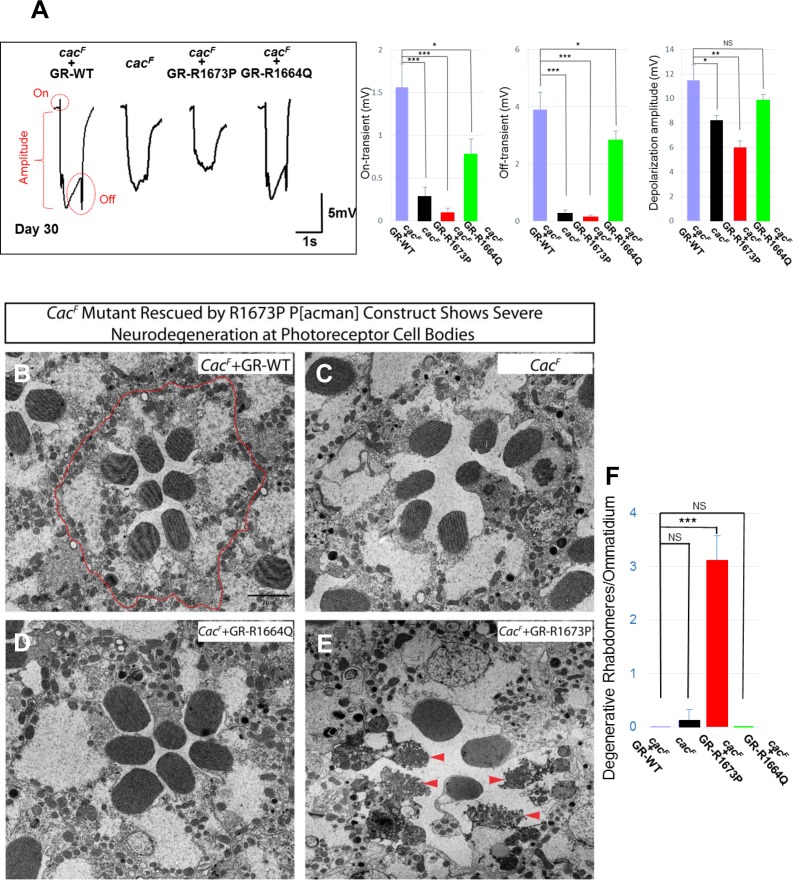
Functional and ultrastructural neurodegeneration in CACNA1A R1673P photoreceptors but not R1664Q. **A)** ERGs of 30-day-old *cac*^*F*^ mutant clones in photoreceptors and *cac*^*F*^ mutants carrying a wild type 80 kb P[acman] genomic rescue transgene (GR-WT) or mutant transgenes (GR-R1673P, GR-R1664Q). ***p<0.001; **p<0.01; *p<0.05; NS, not significant. p-values were consistent with those on one way ANOVA. B) Transmission electron microscopy showing the ultrastructure of *cac*^*F*^ mutant photoreceptor clones carrying a wild type 80 kb P[acman] genomic rescue transgene (GR-WT) with a normal pattern of 7 *Drosophila* photoreceptors per ommatidium. C) *cac*^*F*^ mutant photoreceptor clones with 7 *Drosophila* photoreceptors per ommatidium showing no severe degenerative changes. **D)** *cac*^*F*^ mutant photoreceptor clones carrying an 80 kb P[acman] genomic rescue transgene with a missense change corresponding to the R1664Q missense variant with a normal pattern of 7 Drosophila photoreceptors per ommatidium. **E)** *cac*^*F*^ mutant photoreceptor clones carrying an 80 kb P[acman] genomic rescue transgene with a missense change corresponding to the R1673P missense variant with dramatic neurodegeneration (red arrowheads) involving the photoreceptors. **F)** Quantification of degenerative rhabdomeres per ommatidium. ***p<0.001; NS, not significant. p-values were consistent with those on one way ANOVA.

As increased Ca^2+^ influx typically causes excitotoxicity which may lead to the demise of neurons [6], we recorded the ERGs of 8 genotypes (*cac*^*F*^ or *cac*^*J*^ mutations rescued by GR-WT, GR-1673P, GR-1664Q, or no rescue) in 30-day-old flies. We observed a loss of depolarization amplitude in the R1673P (Patient 1) flies in the *cac*^*F*^ (missense) mutant background (**Fig 4A**). In contrast, GR-R1664Q (Patients 2–5) did not lead to a significant decrease in amplitude. Interestingly, when the transgenes were tested in the *cac*^*J*^ (nonsense) mutant background the reduction in amplitude for R1673P was less severe although still statistically significant (**S2C Fig**). Similar to those tested in *cac*^*F*^ background, the R1664Q variant did not lead to a significant decrease in amplitude (**S2C Fig**). These data suggest that the increase in activity observed in young flies that carry the R1673P variant leads to age-dependent deterioration of the phototransduction pathway, a phenotype that has not been previously observed in *cac* loss-of-function mutations [6].

### Ultrastructure of *Drosophila* photoreceptors with *CACNA1A* alleles

To assess if the R1673P allele may also affect the ultrastructure of the photoreceptors, we performed transmission electron microscopy (TEM). 30-day-old *cac*^*F*^ mutant flies show a slight change in retinal morphology, but the photoreceptor cell bodies retain all seven rhabdomeres and normal overall structure compared to *cac*^*F*^ rescued by the wild type *cac* containing P[acman] clone (**Fig 4C versus 4B**). Moreover, the *cac*^*F*^ mutants rescued with GR-R1664Q (Patients 2–5) did not exhibit obvious morphological defects of photoreceptors at 30 days (**Fig 4D**). In contrast, the *cac*^*F*^ mutant photoreceptors rescued by GR-R1673P (Patient 1) show obvious features of photoreceptor neurodegeneration (**Fig 4E**). The rhabdomeres of the photoreceptors are severely disrupted (arrows), and the cytoplasms of these cells are filled with autophagic vesicles implying neurodegeneration. The severe neurodegenerative phenotype observed here has never been seen in any *cac* alleles, including the null alleles, thus suggesting that R1673P may act via a (toxic) gain-of-function mechanism.

**Fig 5:**
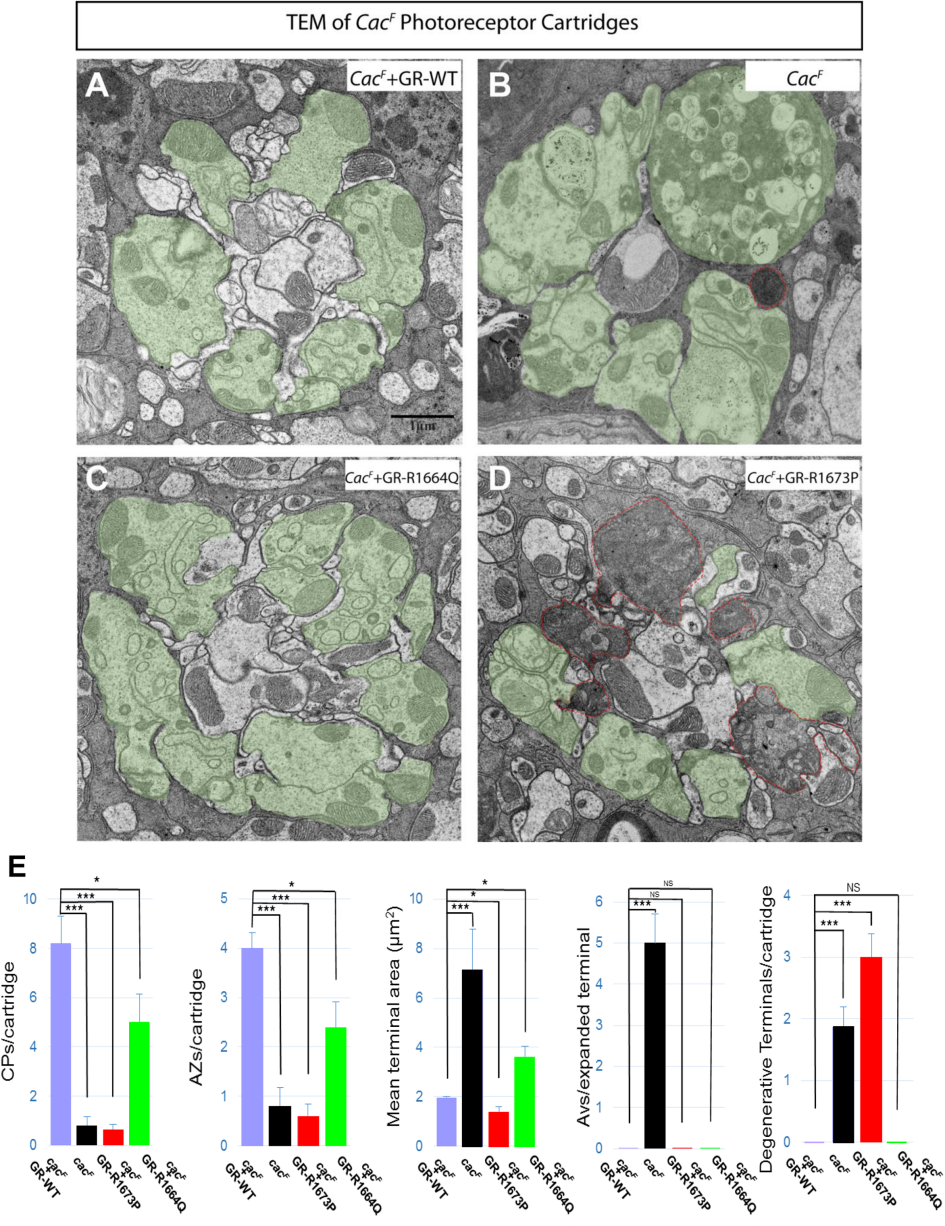
Ultrastructural neurodegeneration in CACNA1A R1673P photoreceptors but not R1664Q in the lamina layer. A) Transmission electron microscopy showing the ultrastructure of *cac*^*F*^ mutant photoreceptor at the level of the lamina where the photoreceptor neurons synapse onto the laminar neurons. Clones carrying a wild type 80 kb P[acman] genomic rescue transgene (GR-WT) have a normal pattern of six *Drosophila* photoreceptors. B) *cac*^*F*^ mutant photoreceptor clones with expansion and degenerative changes. **C)** *cac*^*F*^ mutant photoreceptor clones carrying an 80 kb P[acman] genomic rescue transgene with a missense change corresponding to the R1664Q missense variant with reduced expansion. **D)** *cac*^*F*^ mutant photoreceptor clones carrying an 80 kb P[acman] genomic rescue transgene with a missense change corresponding to the R1673P missense variant with dramatic neurodegeneration evidenced by loss of the normal ultrastructure, and apparent filling with disorganized electron dense material (red outlined areas) involving the synapsing photoreceptors. **E)** Quantification of capitate projections (CPs), active zones (AZs), mean terminal areas, autophagic vesicles per expanded terminal, and degenerative terminals per cartridge, respectively. Data are presented as means ± SEM. p values were calculated using Student’s t test. ***p < 0.001; *p<0.05; NS, not significant. p-values were consistent with those on one way ANOVA for all groups.

We also performed TEM at the level of the lamina, where the presynaptic photoreceptors make synaptic connections with post-synaptic neurons. These data show even more dramatic differences in phenotype between the *cac*^*F*^ mutants rescued by wild type and mutant P[acman] transgenes (**Fig 5**). *cac*^*F*^ rescued by the wild type *cac* P[acman] clone displayed normal morphology of six photoreceptor terminals (green areas in **Fig 5A**). Consistent with our previous findings [6], the *cac*^*F*^ mutant exhibited aberrantly expanded terminals, accumulation of autophagic vacuoles (AVs) and some signs of synaptic degeneration (red area in **Fig 5B**). The P[acman] clone containing the R1664Q (Patients 2–5) variant partially rescued this terminal expansion phenotype (**Fig 5C**), whereas the *cac*^*F*^ mutant rescued by GR-R1673P (Patient 1) shows smaller size of their terminals (**Fig 5D**, Quantification in **Fig 5E**), a phenotype which is likely due to the depletion of more synaptic vesicles with gain of channel function. In addition, we saw evidence of a dramatic synaptic degeneration in the GR-R1673P rescued flies (red outlined areas in **Fig 5D**) compared to the other genotypes. These data indicate that the R1673P mutation causes severe neurodegeneration in both photoreceptor cell bodies and terminals, likely due to gain of channel function that is toxic to neurons during aging.

Interestingly, we did not observe any severe neurodegeneration in *cac*^*J*^ (nonsense) mutants rescued by GR-R1673P in both retinae and laminae (**S3** and **S4 Figs**). Instead, we observed partial rescue of the terminal expansion phenotype, and notably autophagic vacuoles did not accumulate (**S4D Fig**). The latter suggests that lysosome-autophagosome fusion function is still present in *cac* with the R1673P mutation. The differences in the phenotypes observed in *cac*^*F*^ and *cac*^*J*^ mutants rescued by GR-R1673P further support that R1673P is a gain-of-function variant. In the *cac*^*F*^ partial loss-of-function background, the R1673P variant can generate toxic gain-of-function effects that lead to severe neurodegeneration. In contrast, the *cac*^*J*^ null allele expresses no endogenous *cac* and therefore alleviates the toxicity that arises from the R1673P variant. In summary, our experiments in *Drosophila* strongly suggest that the R1673P and R1664Q mutations are likely to be functional in human and likely to exert their effects through distinct mechanisms.

## Discussion

We report five individuals with similar clinical presentations of ataxia, expressive speech delay, motor incoordination, and age of onset. The severe early-onset ataxias seen in these patients are similar to reports of severe early-onset ataxia associated with *CACNA1A* missense variants observed in the S4 transmembrane segment of domain III (e.g. p.I1342T, p.V1396M, and p.R1352Q) [27] or domain IV (R1664Q) [34]. Given these unique clinical features, it had been proposed that these represented loss-of-function mutations of the calcium channel [34]. These severe ataxia phenotypes were thought to represent the most severe end of the spectrum of EA2 rather than the gain-of-function mechanisms seen in hemiplegic migraines [29, 34]. However, in our series, Patient 1, a girl with a *de novo* R1673P variant, also exhibited a progressive cerebellar neurodegenerative process of the most severe end of the *CACNA1A* clinical spectrum.

Our *Drosophila* studies indicate distinct functional consequences when comparing R1673P and R1664Q alleles. Initially we observed the R1673P allele failed to rescue lethality whereas R1664Q partially rescued, suggesting R1673P is a more severe allele. Importantly, the R1673P allele causes a neurodegenerative phenotype based on functional and morphological criteria which was not seen in either R1664Q or in loss-of-function alleles of *cac*. Despite the overall clinical similarity between the patients, the two alleles exhibit dramatic functional differences in *Drosophila*, a functional spectrum not observed previously in severe *CACNA1A* variants. In retrospect, we note that Patient 1, the only subject with the R1673P variant, had progressive neurodegeneration of the cerebellum, which was a distinguishing feature between her and the other four subjects. The molecular mechanism for the special arginine at position 1673 in regulating the Ca^2+^ channel function remains unclear. Interestingly, the recently solved crystal structure of CaV1.1 suggests that both R1664 and R1673 are positively charged residues within the voltage sensor[43]. Since many disease-associated variants are found in positively charged residues in the S4 segment of domain IV of CACNA1A (**Fig 2A’**), to explore their functional differences will be an exciting future topic to eventually establish a mechanistic model for these key residues in Ca^2+^ channel function.

Responsiveness to treatment and medication differs between reported cases of loss-of-function and gain-of-function *CACNA1A* alleles [11, 18]. Patients with EA2 and loss-of-function alleles are often responsive to acetazolamide, while patients with gain-of-function alleles and FHM may respond but generally tend to be less responsive. One would predict that calcium channel blockers might be more effective for gain-of-function alleles. Whether patients with the more severe ataxias also differ clinically in their response to treatment remains to be tested. We note that, while Patient 5 had a strong positive response to acetazolamide, Patient 1 did not respond, consistent with this observation. Indeed as a result of our study Patient 1 was started on a calcium channel blocker. In conclusion, deciphering the functional impact of these severe *CACNA1A* alleles may provide insight into the pathogenic mechanisms and help direct therapeutic interventions.

## Materials and methods

### Human studies and whole exome sequencing

All human subjects research was approved by the Institutional Review Board at Baylor College of Medicine (Studies- Undiagnosed Diseases Network protocol, 15-HG-0130, approved by the National Human Genome Research Institute IRB, and in the Baylor-Hopkins Center for Mendelian Genomics protocol, H-29697, and "Evaluation of Sequence Variants" H-22769 approved by the IRB for Baylor College of Medicine). All families gave written informed consent for whole exome sequencing and publication. All sequencing studies were performed on genomic DNA from blood samples. Patient 1 obtained trio-based exome sequencing through BHCMG [31]. In brief, for patient 1 DNA samples were obtained and prepared into Illumina paired-end libraries and whole-exome capture with BCM-HGSC core design (52 Mb, Roche NimbleGen), and then sequencing on the Illumina HiSeq 2000 platform (Illumina). The produced data were aligned and mapped to the human reference genome (hg19) through the Mercury pipeline [44]. Single-nucleotide variants (SNVs) were called with the ATLAS (an integrative variant analysis pipeline optimized for variant discovery) variant calling method and annotated by the in-house Cassandra annotation pipeline that adapts ANNOVAR (Annotation of Genetic Variants) and additional in-house tools [45–47]. *De novo* variants were calculated by an in-house developed pipeline (DNM-Finder) [31] for in silico subtraction of parental variants from the proband’s variants in vcf files while accounting for the read number information extracted from BAM files. Bioinformatic tools predicted conservation and pathogenicity of candidate variants, and variants were compared against both an internal database and public databases such as the Exome Aggregation Consortium (ExAC) database.

Patients 2–5 had clinical proband exome sequencing at BGL [32, 33]

### Fly genetics

*cac*^*F*^ and *cac*^*J*^ mutants were isolated from a chemical mutagenesis screen as described previously [6, 48]. The mapping and sequencing of the mutants was performed as described [49]. The P[acman] BAC construct that contains the full length *cac* genomic region was selected from a large P[acman] library that we previously described [49]. A Transgenic line from this BAC (CH321-60D21) was generated previously and named *Dp(1;3)DC131* [49] which we refer to as GR-WT in this paper.

Two point mutations (R1664Q or R1673P) were introduced into the CH321-60D21 BAC by recombineering using the modified *DH10B* strain *SW102* and a *galK* positive/counter selection cassette [50]. The reagents for recombineering were obtained from Biological Resources Branch at National Cancer Institute (NCI) -Frederick. The microinjections to generate transgenic flies that contain wild type or mutant 77kb P[acman] clones were performed by GenetiVision, Houston, TX.

Lethality rescue experiments in *Drosophila* were done by crossing *y w*, *cac*^*F*^ *FRT19A/FM7c or y w*, *cac*^*J*^ *FRT19A/FM7c* with *y w/Y; Dp(1;3)DC131(or -R1673P or -R1664Q) flies*. The males that have no *FM7c* marker in the next generation were considered rescued flies. The numbers of rescued flies were also compared with *FM7c* males in the same progeny.

### Generation of *cac* mutant eye clones in *Drosophila*

In order to circumvent the lethal phenotype, we generated mosaic clones in the *Drosophila* eyes for ERG and EM experiments. Virgin females of *y w*, *cac*^*F*^ (or *cac*^*J*^) *FRT19A* / *FM7c*; *Dp(1;3)DC131-R1673P* (or +) / *CyO* were crossed with cl(1)* FRT19A/ Y; ey-FLP males and we examined *y w*, *cac*^*F*^ (or *cac*^*J*^) FRT19A / cl(1)* FRT19A; ey-FLP/ Dp(1;3)DC131-R1673P (or +) progeny for ERG and EM defects. Homozygous mutant cells were marked by *w*^*-*^ and heterozygous cells were marked by *w*^*+*^. Homozygous wild-type cells were eliminated by the recessive cell lethal mutation (cl(1)*) to give the mutant clones a growth advantage.

### Electroretinogram (ERG) recording

ERG recordings were performed as previously described [51]. Briefly, adult flies were glued to a glass slide, a recording electrode was placed on the surface of the eye while a reference probe was inserted in the thorax. A fly eye was exposed to a flash of white light for 1 second. Responses were recorded and analyzed with AXON ^TM^-pCLAMP8 software. Data were analyzed by two-tailed unpaired Student’s t test. A p-value of <0.05 was considered statistically significant.

### Transmission electron microscopy (TEM)

Laminae in adult flies were processed for TEM imaging as described [52]. Samples were processed using a Ted Pella Bio-Wave microwave oven with vacuum attachment. Adult fly heads were dissected at 25°C in 4% paraformaldehyde, 2% glutaraldehyde, and 0.1 M sodium cacodylate (pH 7.2). Samples were subsequently fixed at 4°C for 48 hours. 1% osmium tetroxide was used for secondary fixation and subsequently dehydrated in ethanol and propylene oxide, and then embedded in Embed-812 resin (Electron Microscopy Science, Hatfield, PA). 50 nm ultra-thin sections were obtained with a Leica UC7 microtome and collected on Formvar-coated copper grids (Electron Microscopy Science, Hatfield, PA). Specimens were stained with 1% uranyl acetate and 2.5% lead citrate and imaged using a JEOL JEM 1010 transmission electron microscope with an AMT XR-16 mid-mount 16 megapixel CCD camera.

### *Drosophila* genetics

The genotypes of the fly strains used are as follows:

*cac^J^:* *y w cac^J^ FRT19A/ P{w+} cl(1) FRT19A; eyFLP/+*
*cac^F^: y w, cac^F^* *FRT19A/ P{w+} cl(1) FRT19A; eyFLP/+*
*cac^J^ + GR-WT: y w cac^J^ FRT19A/Y;* *Dp(1;3)DC131/+*
*cac^F^ + GR-WT: y w cac^F^ FRT19A/Y; Dp(1;3)DC131/+*,
*cac^J^ + GR-R1673P: y w, cac^J^ FRT19A/ P{w+} cl(1) FRT19A; Dp(1;3)DC131-R1673P/eyFLP*,
*cac^F^ + GR-R1673P: y w, cac^F^ FRT19A/ P{w+} cl(1) FRT19A; Dp(1;3)DC131-R1673P/eyFLP*,
*cac^J^ + GR-R1664Q: y w, cac^J^ FRT19A/Y; Dp(1;3)DC131-R1664Q/+*,
*cac^F^ + GR-R1664Q: y w, cac^F^ FRT19A/Y; Dp(1;3)DC131-R1664Q /+*.

## Supporting information

S1 Case Histories Clinical summaries for patients 1–5. (DOCX)

S1 Table Members of the Undiagnosed Diseases Network. The members of the UDN listed.
(XLSX)

S1 Fig Imaging and molecular characteristics of the patients with *CACNA1A de novo* variants. A) Patient 4 brain MRI at 8 years showing a mild atrophy of the cerebellar vermis. B) Patient 4 brain MRI axial image showing normal cerebellar hemispheres. C) All five subjects had *de novo* missense variants in *CACNA1A*. Sanger traces for each father, mother and proband are shown below, the red arrows indicate the position of the *de novo* variant.
(TIF)

S2 Fig Electroretinogram recordings from the *cac*^*J*^ alleles rescued by the P[acman] constructs. **A)** ERGs of 3-day-old *cac*^*J*^ mutant clones in photoreceptors and *cac*^*J*^ mutants carrying a wild type 80 kb P[acman] genomic rescue transgene (GR-WT) or mutant transgenes (GR-R1673P, GR-R1664Q). Similar results to those were seen in the *cac*^*F*^ background in **Fig 2E.** B) ERGs of 30-day-old *cac*^*J*^ mutant clones in photoreceptors and *cac*^*J*^ mutants carrying a wild type 80 kb P[acman] genomic rescue transgene (GR-WT) or mutant transgenes (GR-R1673P, GR-R1664Q). ***p<0.001; *p<0.05; NS, not significant. *, p < 0.05, one-way ANOVA.
(TIF)

S3 Fig TEM of *cac*^*J*^ photoreceptors in retinae shows no severe neurodegeneration in CACNA1A R1673P. **A.** Transmission electron microscopy showing the ultrastructure of *cac*^*J*^ mutant photoreceptor clones carrying a wild type 80 kb P[acman] genomic rescue transgene (GR-WT) with a normal pattern of 7 *Drosophila* photoreceptors per ommatidium. **B.** *cac*^*J*^ mutant photoreceptor clones with 7 *Drosophila* photoreceptors per ommatidium showing no severe degenerative changes. **C.** *cac*^*J*^ mutant photoreceptor clones carrying an 80 kb P[acman] genomic rescue transgene with a missense change corresponding to the R1664Q missense variant with a normal pattern of 7 Drosophila photoreceptors per ommatidium. **D.** *cac*^*J*^ mutant photoreceptor clones carrying an 80 kb P[acman] genomic rescue transgene with a missense change corresponding to the R1673P missense variant with mild neurodegeneration involving the photoreceptors.
(TIF)

S4 Fig TEM of *cac*^*J*^ photoreceptor cartridge. **A.** Transmission electron microscopy showing the ultrastructure of *cac*^*J*^ mutant photoreceptor at the level of the lamina where the photoreceptor neurons synapse onto the laminar neurons. Clones carrying a wild type 80 kb P[acman] genomic rescue transgene (GR-WT) have a normal pattern of six Drosophila photoreceptors. **B.** *cac*^*J*^ mutant photoreceptor clones with severe expansion and accumulation of autophagic vesicles. **C.** *cac*^*J*^ mutant photoreceptor clones carrying an 80 kb P[acman] genomic rescue transgene with a missense change corresponding to the R1664Q missense variant show reduced expansion. **D.** *cac*^*J*^ mutant photoreceptor clones carrying an 80 kb P[acman] genomic rescue transgene with a missense change corresponding to the R1673P missense variant partially rescued *cac*^*J*^ phenotypes. **E**. Quantification of capitate projections (CPs), active zones (AZs), mean terminal areas, autophagic vesicles per expanded terminal, and degenerative terminals per cartridge, respectively. Data are presented as means ± SEM. p values were calculated using Student’s t test. ***p < 0.001; **p<0.01; *p<0.05; NS, not significant. ***, p < 0.001, one-way ANOVA for all groups.
(TIF)
