# Supplementary material for: Clinically severe CACNA1A alleles affect synaptic function and neurodegeneration differentially
Source: PLoS Genet. 2017 Jul 24;13(7):e1006905. doi: 10.1371/journal.pgen.1006905 (PMC5557584; doi:10.1371/journal.pgen.1006905)
Supplement: S1 Case Histories — (DOCX) [file pgen.1006905.s001.docx]

**Supplemental Clinical Histories**

***Patient 1: de novo (NM_001127221.1:c.5018G>C: p.R1673P; chr19:13346480C>G [hg19]) variant in CACNA1A.***

Patient 1 was enrolled at age 8 years in the Undiagnosed Diseases Network (UDN) at Baylor College of Medicine and concurrently in the Baylor-Hopkins Centers for Mendelian Genomics (BHCMG) for a long-standing history of global developmental delay, hypotonia and progressive cerebellar degeneration. She underwent trio exome sequencing through BHCMG. She was also enrolled in the UDN at Baylor College of Medicine for research clinical evaluation at Texas Children’s Hospital that included neurological, medical genetic, ophthalmologic, and neuroimaging evaluations.

She was the product of an uncomplicated pregnancy from non-consanguinous parents, born to a 38-year-old G4P3-4 mother, birth weight 6lb 13oz. She was born by spontaneous vaginal delivery, had a nuchal cord and was cyanotic initially but recovered such that Apgar scores were 4 at one minute and 9 at 5 minutes. Concerns were first raised for developmental delay at 4 months of age due to poor head control. She was started on physical and occupational therapy. She had motor incoordination and communication impairment with a severe impairment in expressive language but sparing receptive language and eventually learned signs and was able to use an iPad. She has experienced no appreciable developmental regression. At her evaluation at age 8 she was noted to have ataxia and motor incoordination with need for a walker, although she had no truncal ataxia and no tibubation. She was nonverbal. Her eye exam was significant for accommodative esotropia. Her growth parameters including head circumference were age-appropriate. The neurological exam was pertinent for central hypotonia and reduced deep tendon reflexes.

Her initial workup for hypotonia included normal urine organic acids, plasma amino acids, acylcarnitine profile, creatine guanidinoacetate, very long chain fatty acids, *MECP2* sequencing, and array comparative genomic hybridization (aCGH). She also underwent sequencing studies for a number of single gene disorders including Spinocerebellar Ataxia 8 (SCA8), SCA10, SCA17, *DRPLA*, *FRDA1, APTX, SCA14, SETX, POLG1, SCA5, SIL1, TTPA, KCNC3, NPC1, NPC2* none of which provided a diagnosis. She had a muscle biopsy that showed increased oxidative enzyme activity, while mitochondrial mutation analysis and respiratory chain activity were normal.

Neuroimaging for the patient was significant for progressive cerebellar atrophy. While the initial MRI at 10 months showed a normal cerebellum (**Figure 1A**), imaging at 22 months revealed mild cerebellar atrophy (**Figure 1B**), which progressed at 3.5 years (**Figure 1C**) and 8 years (**Figure 1D**). She had bilateral medial rectus surgery for accommodative esotropia.

Exome sequencing revealed a novel missense variant in *CACNA1A*, which was not seen in ClinVar, ExAC, nor in local databases. The variant was found to be *de novo*.

***Patient 2: de novo (NM_001127221.1:c.4991G>A: p.R1664Q; chr19:13346507C>T [hg19]) variant in CACNA1A***

Patient 2 presented in infancy with global developmental delay and language delay. She underwent whole-exome sequencing at Baylor Genetics.

She was the product of an uncomplicated pregnancy from non-consanguinous parents, born to her then 17-year-old G1P0 mother and 20-year-old father. She was carried to 42 weeks born induced, weighing 7 pounds and 6 ounces with a length of 21.5 inches. She was kept in the hospital for 3 days and treated for hyperbilirubinemia. At her initial evaluation at 2 years, 9 months she was noted to have ataxia and hyperreflexia. According to the mother, there had always been some degree of developmental delay with slow attainment of milestones.

At the time of her last evaluation at age 5 years, she was found to have a severe ataxia with difficulty in achieving independent ambulation. She had very few words and continued to have global developmental delay. She had worsening behavioral problems and was noted to have hyperreflexia. She was also noted to have disordered eye movements.

Prior workup included creatine kinase, lipid panel, creatine/guanidinoacetate, amino acids, carnitine/acylcarnitine combo, organic acids, acylglycines, carbohydrate deficient transferrin, plasma ammonia, O-glycans, very long chain fatty acids, and GAA repeat expansion. She had a normal SNP microarray. She underwent a muscle biopsy and results of the electron transport chain and quantitated PCR of nuclear and mitochondrial genes were normal. Histopathology was essentially normal also. Cerebrospinal fluid obtained at the same time as the muscle biopsy revealed normal amino acids and normal neurotransmitters.

Brain MRI was performed at age 1 year, 8 months and was normal with a normal appearance of the cerebellum.

Whole-exome sequencing revealed a missense variant in *CACNA1A*, the variant, not seen in ExAC was found to be *de novo* and was identified in four other patients in this series.

***Patient 3: de novo (NM_001127221.1:c.4991G>A: p.R1664Q; chr19:13346507C>T [hg19]) variant in CACNA1A***

Patient 3 presented in infancy with “hypotonic cerebral palsy” and language delay. He walked at 27 months and had no speech until 15 months He underwent clinical exome sequencing at Baylor Genetics.

He was the product of an uncomplicated pregnancy from non-consanguinous parents, born to a 30-year-old G2P1-2 mother, birth weight 8lb 9oz. He was born by induced vaginal delivery and had a normal neonatal course. Concerns were first raised in the first year of life for delay in his acquisition of motor milestones. He did not begin crawling until 10 months of age and walked at 27 months of age. At his initial evaluation at 30 months of age, he had been described to have a wide-based gait, with a tendency to fall and bruise himself very frequently. Body tone was also noted to be low, and he had chronic problems with recurrent GE reflux, ear infections and possible brief absence seizures.

He was diagnosed with “hypotonic cerebral palsy” and congenital ataxia. He had long-standing non progressive motor incoordination. He also had issues with urinary incontinence.

At the time of his last evaluation at age 5 years he was found on examination to have a wide-based gait, significant toe walking, need for AFOs with concern for bilateral ankle tightness and requirement for an adaptive stroller for long distances. He had developed a lot of issues with difficulty sleeping, and sensory processing disorders. He was able to sit and walk independently and participated in swimming. His speech was dysarthric. His ophthalmologic evaluation was significant for strabismus, myopia and astigmatism.

Prior workup included normal urine organic acids, plasma amino acids, lactate, creatine kinase, very long chain fatty acids, screening for congenital disorders of glycosylation, urine and serum guanidinoacetate profile, DNA methylation for Prader-Willi/Angelman and fragile X syndromes, *PTEN* and *HRAS* gene sequencing, and array comparative genomic hybridization (aCGH).

Brain MRI was performed at age 1 year and 5 months (**Figure 1E**) and showed a thin corpus callosum posteriorly with a normal appearance of the cerebellum.

Exome sequencing revealed a missense variant in *CACNA1A*, which was not seen in ExAC, found to be *de novo*, and identified in four other patients in this series.

***Patient 4: de novo (NM_001127221.1:c.4991G>A: p.R1664Q; chr19:13346507C>T [hg19]) variant in CACNA1A***

Patient 4 presented in infancy with hypotonia, global developmental delay and language delay. He had a long-standing history of hypotonia, dysarthric speech and cognitive delay. He underwent clinical exome sequencing at Baylor Genetics.

He was the product of pregnancy complicated by multiple respiratory infections and maternal smoking from non-consanguinous parents. He was born at 40 weeks with birth weight of 7lb 14oz. In the neonatal period he developed hyperbilirubinemia and was treated with phototherapy for 3 days. He sat at 9 months, pulled to stand at 13-14 months, started cruising at 21 months and was walking independently at 2 years 6 months. He said his first word at 18 months, and at 2 years he could say 5 words.

At the time of his last evaluation at age 8 years he was found to have a wide-based and unsteady gait. On examination he was found to have inverted nipples. He has difficulty following commands, ocular apraxia, no nystagmus, drooling, dysarthria, hypotonia, ataxic gait, slow rapid alternating movements, bilateral dysmetria, and no tremors. He had developed issues with aggressive behavior where frustration would lead to episodes at least 3 times a week. He was in regular 2^nd^ grade and in resource classes for reading and math with an IQ just below normal. He was receiving PT, OT and speech therapy.

Brain MRI was performed at 8 years of age. The sagittal T1 (**Figure S1A**) shows mild atrophy of the cerebellar vermis with normal brain stem and corpus callosum. The axial T1 shows the normal cerebellar hemispheres (**Figure S1B**).  Axial T2 Flair showed normal basal ganglia, ventricular system, cerebral cortex and cerebral white matter.

Exome sequencing revealed a missense variant in *CACNA1A*, which was not seen in ExAC, found to be *de novo*, and identified in four other patients in this series.

***Patient 5: de novo (NM_001127221.1:c.4991G>A: p.R1664Q; chr19:13346507C>T [hg19]) variant in CACNA1A***

Patient 5 presented in infancy with global developmental delay and language delay. She had a long-standing history of hypotonia, developmental delay and poor motor coordination. She underwent clinical exome sequencing at Baylor Genetics.

She was the product of an unremarkable pregnancy from non-consanguinous parents. She had a prenatal chromosomal microarray for the indication of advanced maternal age, and the results were normal. The delivery was uneventful.

She was a healthy infant but was noted by her parents to have some vertigo in the sense of fear of being held upside down during play or riding on a parents shoulders. She had gross motor delay and had an extensive workup starting at 18 months of age. After exome sequencing revealed the *CACNA1A* variant the child was put on a trial of acetazolamide and showed near immediate dramatic improvement in balance. At the time of her last evaluation at age 7 years she was found to have a wide-based and unsteady gait with poor coordination, truncal ataxia a hip sway and a speech and language disorder. She was dysarthric and dysgrammatic. Her ophthalmologic evaluation was significant for strabismus and esotropia. She was taking Ritalin, Vyvanse, Vitamin E, Diamox and a selective serotonin reuptake inhibitor (SSRI).

Prior workup included mitochondrial DNA sequencing studies and normal microarray. She had EMG and nerve conduction studies, which were normal. She had CSF amino acids, which suggested a dysfunction in serotonin metabolites.

Brain MRI was performed at ages 24 months (**Figure 1F**) and 46 months, both normal.

Exome sequencing revealed a missense variant in *CACNA1A*, which was not seen in ExAC, found to be *de novo*, and identified in four other patients in this series.
