# Supplementary figures and images for: Clinically severe CACNA1A alleles affect synaptic function and neurodegeneration differentially
Source: PLoS Genet. 2017 Jul 24;13(7):e1006905. doi: 10.1371/journal.pgen.1006905 (PMC5557584; doi:10.1371/journal.pgen.1006905)

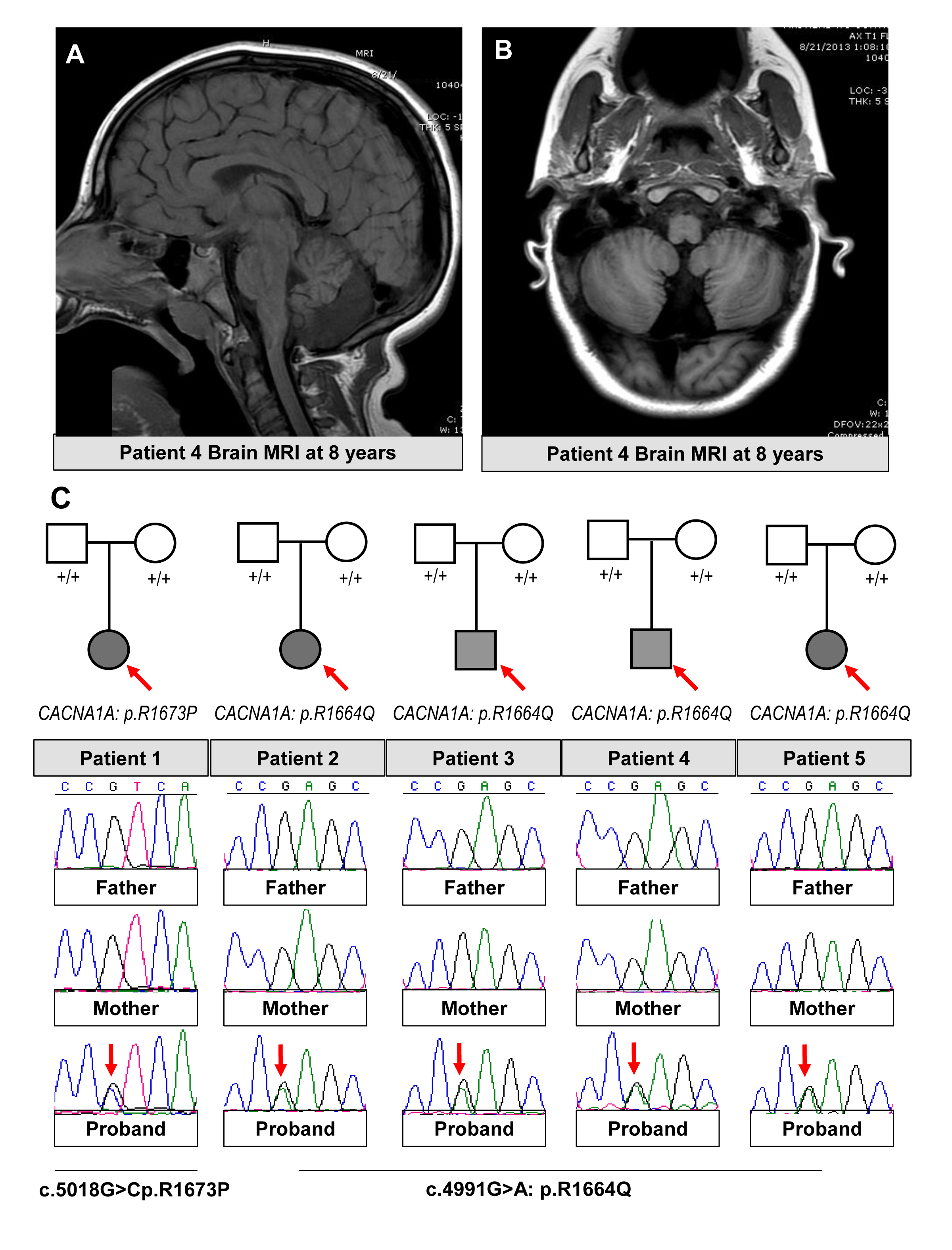

Supplement: S1 Fig — A) Patient 4 brain MRI at 8 years showing a mild atrophy of the cerebellar vermis. B) Patient 4 brain MRI axial image showing normal cerebellar hemispheres. C) All five subjects had de novo missense variants in CACNA1A. Sanger traces for each father, mother and proband are shown below, the red arrows indicate the position of the de novo variant. (TIF) [file pgen.1006905.s003.tif]

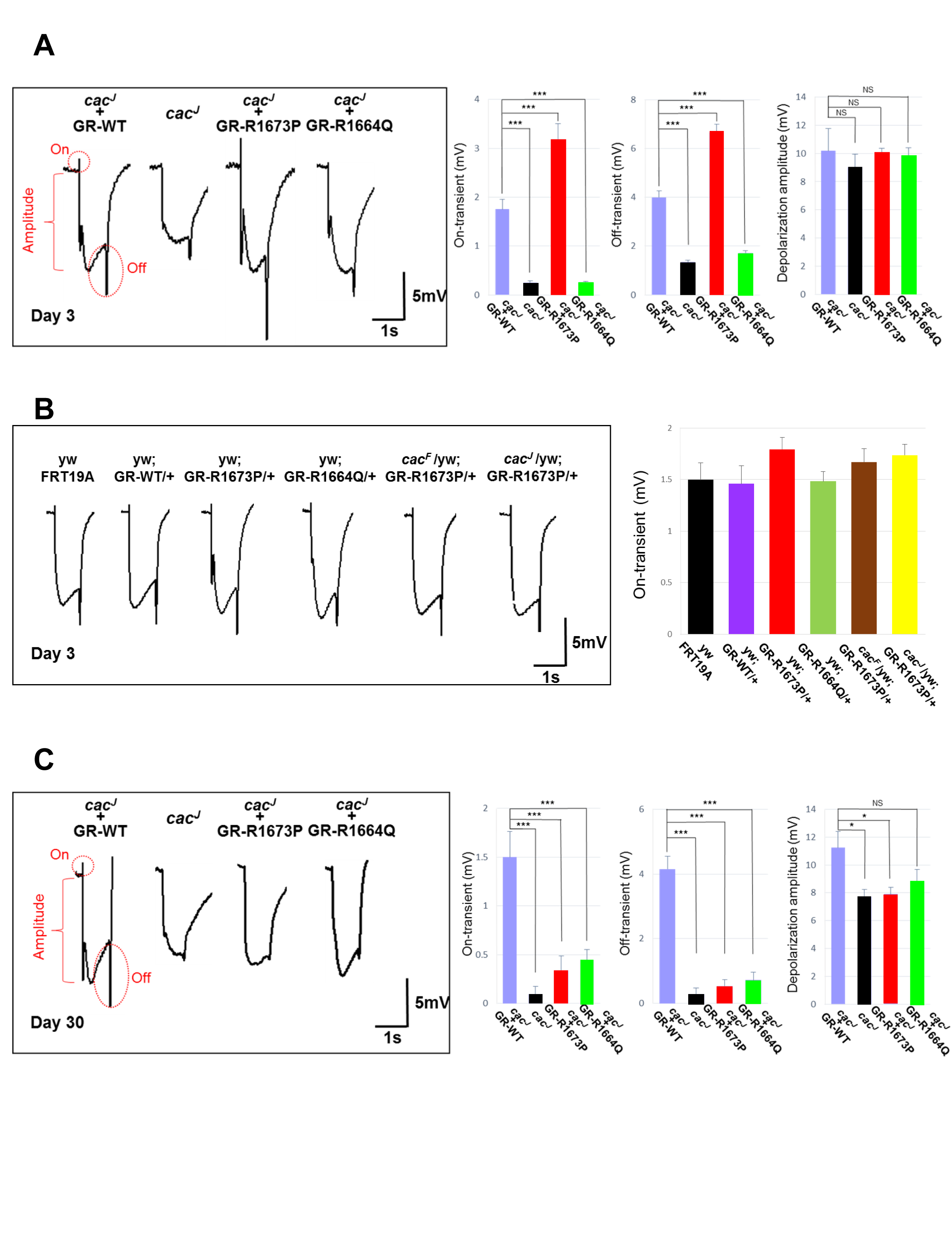

Supplement: S2 Fig — A) ERGs of 3-day-old cacJ mutant clones in photoreceptors and cacJ mutants carrying a wild type 80 kb P[acman] genomic rescue transgene (GR-WT) or mutant transgenes (GR-R1673P, GR-R1664Q). Similar results to those were seen in the cacF background in Fig 2E. B) ERGs of 30-day-old cacJ mutant clones in photoreceptors and cacJ mutants carrying a wild type 80 kb P[acman] genomic rescue transgene (GR-WT) or mutant transgenes (GR-R1673P, GR-R1664Q). ***p<0.001; *p<0.05; NS, not significant. *, p < 0.05, one-way ANOVA. (TIF) [file pgen.1006905.s004.tif]

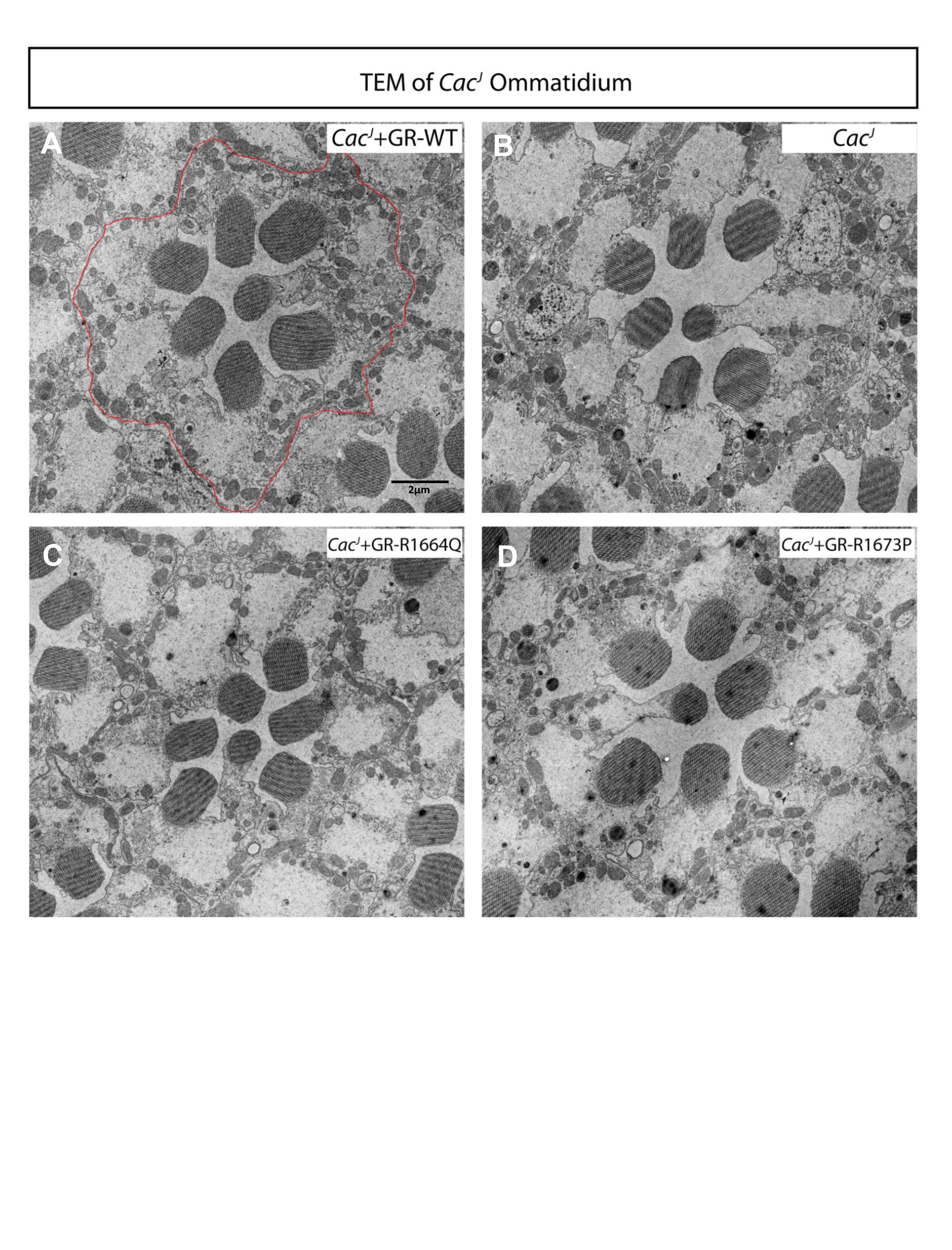

Supplement: S3 Fig — A. Transmission electron microscopy showing the ultrastructure of cacJ mutant photoreceptor clones carrying a wild type 80 kb P[acman] genomic rescue transgene (GR-WT) with a normal pattern of 7 Drosophila photoreceptors per ommatidium. B. cacJ mutant photoreceptor clones with 7 Drosophila photoreceptors per ommatidium showing no severe degenerative changes. C. cacJ mutant photoreceptor clones carrying an 80 kb P[acman] genomic rescue transgene with a missense change corresponding to the R1664Q missense variant with a normal pattern of 7 Drosophila photoreceptors per ommatidium. D. cacJ mutant photoreceptor clones carrying an 80 kb P[acman] genomic rescue transgene with a missense change corresponding to the R1673P missense variant with mild neurodegeneration involving the photoreceptors. (TIF) [file pgen.1006905.s005.tif]

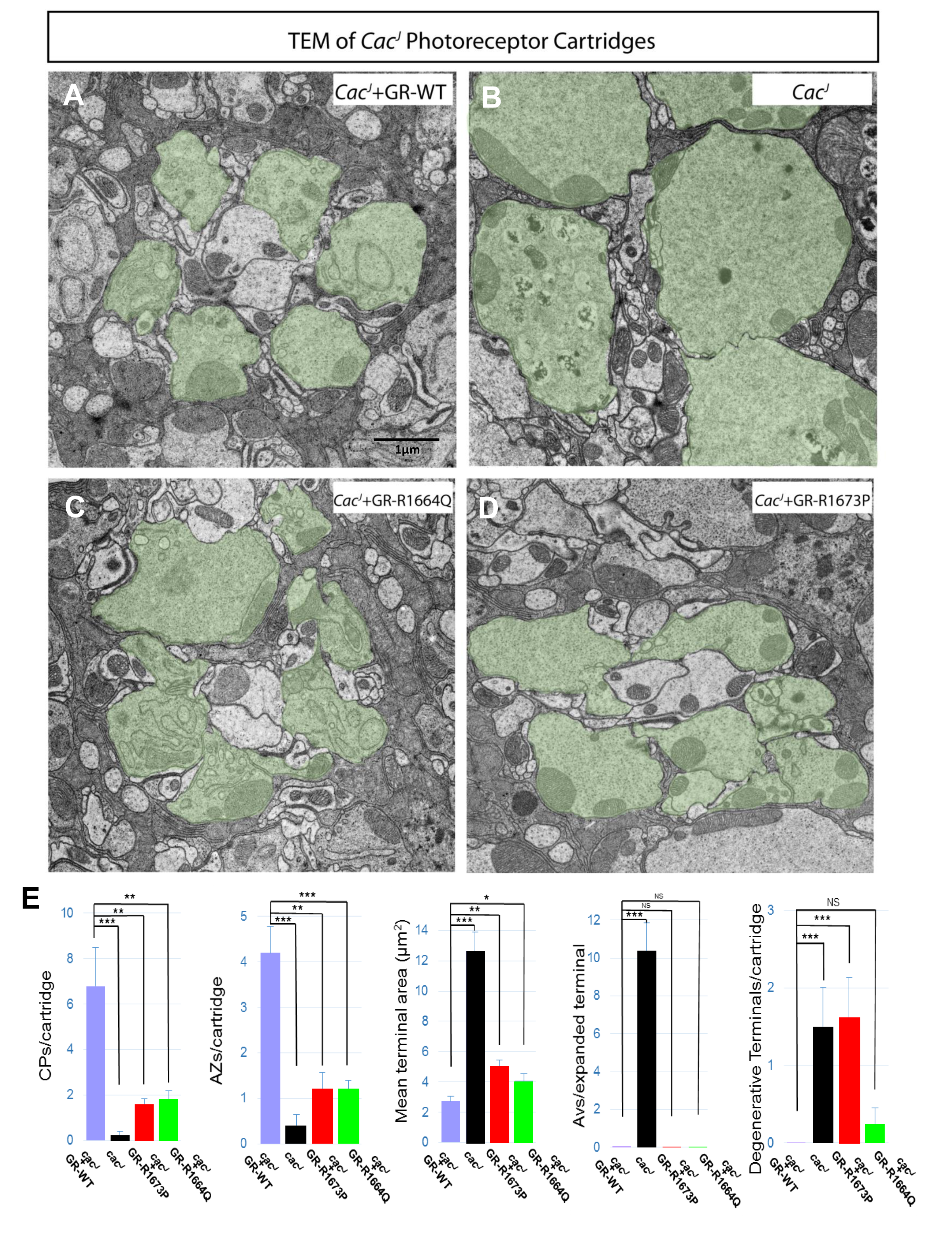

Supplement: S4 Fig — A. Transmission electron microscopy showing the ultrastructure of cacJ mutant photoreceptor at the level of the lamina where the photoreceptor neurons synapse onto the laminar neurons. Clones carrying a wild type 80 kb P[acman] genomic rescue transgene (GR-WT) have a normal pattern of six Drosophila photoreceptors. B. cacJ mutant photoreceptor clones with severe expansion and accumulation of autophagic vesicles. C. cacJ mutant photoreceptor clones carrying an 80 kb P[acman] genomic rescue transgene with a missense change corresponding to the R1664Q missense variant show reduced expansion. D. cacJ mutant photoreceptor clones carrying an 80 kb P[acman] genomic rescue transgene with a missense change corresponding to the R1673P missense variant partially rescued cacJ phenotypes. E. Quantification of capitate projections (CPs), active zones (AZs), mean terminal areas, autophagic vesicles per expanded terminal, and degenerative terminals per cartridge, respectively. Data are presented as means ± SEM. p values were calculated using Student’s t test. ***p < 0.001; **p<0.01; *p<0.05; NS, not significant. ***, p < 0.001, one-way ANOVA for all groups. (TIF) [file pgen.1006905.s006.tif]
